# A Relationship-Based Resilience Program for Promotores: Protocol for a Randomized Controlled Waitlist Trial

**DOI:** 10.2196/51427

**Published:** 2023-12-19

**Authors:** Daniela Arcos, Lyric N Russo, Kelly F M Kazmierski, Elayne Zhou, Gloria Itzel Montiel, America Bracho, Nancy Mejia, Jessica L Borelli

**Affiliations:** 1 Department of Psychological Science University of California Irvine Irvine, CA United States; 2 Department of Psychology University of Southern California Los Angeles, CA United States; 3 Latino Health Access Santa Ana, CA United States

**Keywords:** psychosocial intervention, relational savoring, cardiometabolic health, community service providers, Hispanic or Latinx, CSP

## Abstract

**Background:**

Community service providers (CSPs) play an integral role in the health care of low-income Hispanic or Latinx (HL) communities. CSPs have high-stress frontline jobs and share the high-risk demographics of their communities. Relational savoring (RS) has been associated with lower cardiovascular reactivity and psychosocial benefits, with particular promise among HL participants. In this study, we aim to identify RS’s potential in promoting CSPs’ cardiometabolic health and, in so doing, having broader impacts on the community they serve.

**Objective:**

This randomized controlled waitlist study aims to examine the effect of an RS intervention on (1) CSPs’ cardiometabolic health (cardiometabolic risk factors and outcomes) and (2) CSPs’ threats to leaving the workforce.

**Methods:**

We will recruit a sample of 80 CSPs from community health agencies serving low-income HL populations. Participating CSPs will be randomized into an experimental or a waitlist control. Participants will complete 1 or 2 baseline assessment batteries (before the intervention), depending on the assigned group, and then complete 2 more assessment batteries following the 4-week RS intervention (after the intervention and at a 3-mo follow-up). The RS intervention consists of guided reflections on positive moments of connection with others. Electrocardiogram data will be obtained from a wearable device (Polar Verity Sense or Movisens) to measure heart rate variability. The primary outcome is cardiometabolic health, consisting of cardiometabolic risk (obtained from heart rate variability) and cardiometabolic health behaviors. The secondary outcomes include CSPs’ threats to leaving the workforce (assessed via psychological well-being), intervention acceptability, and CSPs’ delivery of cardiometabolic health programming to the community (exploratory). Analyses of covariance will be used to examine the effects of RS on cardiometabolic health and on CSPs’ threats to leaving the workforce, comparing outcomes at baseline, postintervention, and at follow-up across participants in the experimental versus waitlist group.

**Results:**

The study has been approved by the University of California, Irvine, Institutional Review Board and is currently in the data collection phase. By May 2023, 37 HL CSPs have been recruited: 34 have completed the baseline assessment, 28 have completed the 4 intervention sessions, 27 have completed the posttreatment assessment, and 10 have completed all assessments (including the 3-mo follow-up).

**Conclusions:**

This study will provide valuable information on the potential of RS to support cardiometabolic health in HL CSPs and, indirectly, in the communities they serve.

**Trial Registration:**

ClinicalTrials.gov NCT05560893; https://clinicaltrials.gov/study/NCT05560893

**International Registered Report Identifier (IRRID):**

DERR1-10.2196/51427

## Introduction

### Background

Ethnic and racial disparities in cardiometabolic health within the United States have been widely documented [1-5]. Racial-ethnic minoritized and historically underrepresented groups, including Hispanic or Latinx (HL) Americans, face higher cardiometabolic risks, such as obesity, diabetes, hypertension, and dyslipidemia, than non-HL White Americans [6,7]. According to data obtained from the National Health Interview Survey of 2018, HL adults are 1.3 times more likely to die from diabetes and 70% more likely to be diagnosed with diabetes by a physician than non-Hispanic White (NHW) adults [8]. Similarly, HL Americans are 1.2 times more likely to be obese than NHW Americans, whereas HL women are 20% more likely to be overweight than their NHW counterparts [9]. The etiology of cardiometabolic risk disparities in racial-ethnic minoritized communities, including low-income HL communities, is multifactorial and includes socioeconomic, behavioral, and environmental factors. Cultural and language barriers, lack of health care access, and differences in education and employment opportunities contribute to these disparities [1,5,10]. Community service providers (CSPs), also called *promotores de salud*, are members of the community who provide culturally appropriate health education and information, offer support and care, and advocate for the community’s health needs [11]. These roles empower HL CSPs to help community members overcome or withstand the barriers that contribute to health inequalities.

Through culturally accessible programming, CSPs help prevent and manage chronic diseases, including cardiometabolic diseases, in the communities they serve. Indeed, CSPs play a significant role in reducing health risks and disease burden as well as in the management of various health problems, including hypertension, diabetes, HIV infection, cancer, and cardiovascular disease [12-16]. Community interventions led by CSPs have been proven to effectively lower cardiometabolic risk factors in HL communities while being affordable and well received by community members [12-16]. These include, among others, prevention programs targeting diabetes and obesity, which effectively impact risk factors such as blood pressure, low-density lipoprotein cholesterol, triglycerides, insulin, sedentary behaviors, and one’s weight [12-16]. There are many advantages to CSPs belonging to the same communities they serve, including cultural and linguistic understanding, trust, credibility, and rapport. All of this contributes to community members’ engagement and retention in interventions that improve health outcomes and provide long-term support. However, sharing the same demographic characteristics also means that HL CSPs face the same high cardiometabolic risks as the community members they serve. In fact, it is often the case that CSPs are individuals who themselves have experienced health challenges and have learned to manage their conditions (eg, diabetes, HIV). In addition to providing support to others, as they have been trained to, their own health concerns might still require monitoring and self-management. Furthermore, the high-stress nature of their occupations may further amplify health risks. This is especially true when considering the extensive stress that the COVID-19 pandemic brought to frontline health care workers, including CSPs. In support of this assertion, a qualitative analysis found that the pandemic affected CSPs’ work and daily life, leading to psychological and social stress [17].

CSPs, along with all frontline health care professionals (HCPs) during the pandemic, experienced acute psychological distress and associated effects [17]. A study analyzing the role of CSPs in the COVID-19 pandemic response reports that CSPs faced significant exposure to stress and grief, among other difficulties [18]. In addition, HL communities, as well as other minoritized communities, were more likely to be exposed to the SARS-CoV-2 virus, to experience severe forms of COVID-19, and to face higher mortality rates [19,20]. Understandably, this led to catastrophic financial and social consequences in low-income HL communities. Owing to the comprehensive support that CSPs provide to community members, which addresses not only physical health but also various financial and social needs (eg, unemployment, housing insecurity, food insecurity, domestic violence, and language barriers to navigate legal paperwork), CSPs carried a significant burden [17]. Considering the fact that they are at higher risk for negative health outcomes (eg, diabetes, high blood pressure, and overweight) based on demographics alone and the role they played during the pandemic, which led to heavy workloads and high stress, CSPs may face substantial risk for cardiometabolic disease. Nevertheless, to the best of our knowledge, there have been no interventions aimed at protecting cardiometabolic health specifically in CSPs.

By placing enormous demands on CSPs, the aftereffects of COVID-19 have placed CSPs at risk of professional burnout, endangering their abilities to deliver this care [17,21]. CSPs play a crucial role in the health of underserved communities, but to carry out their greatly needed service, there must be a system that promotes and protects their health and well-being. Supporting CSPs is a matter of equity; in response to the health care system failure to adequately support medically underserved communities, the CSP model represents these communities’ efforts to promote health and build resilience. Although no previous studies have specifically examined how CSPs’ physical and psychological well-being relates to the quality and reach of their services, researchers have investigated how burnout in other HCPs affects the quality and safety of the care they provide [22,23]. In line with theory, a meta-analysis comprising 210,669 HCPs concluded that burnout (depersonalization, emotional exhaustion, and reduced personal accomplishment) is negatively associated with quality (perceived quality and patient satisfaction) and safety of care [22]. Similarly, in a systematic literature review, burnout among physicians was associated with decreased productivity, as evidenced by the number of sick days, hours worked, number of patients seen, and intent to leave the profession [23]. Considering these relationships between HCPs’ burnout and reduced productivity, impaired quality of care, and reduced safety of the care provided, it is reasonable to expect similar associations between CSPs’ well-being and the reach and quality of the services delivered to the community. As such, interventions designed to promote CSPs’ well-being and prevent their burnout are a necessity.

### Relational Savoring: A Culturally Congruent Approach

Relational savoring (RS), based on positive psychology and attachment theory, is a brief intervention in which one deeply reflects on a positive emotional experience involving a moment of connection with another person [24]. The underlying premise is that focusing on a memory of positive connection will augment the meaning and positive emotion associated with this interpersonal experience [24]. Through this process, one’s sense of psychological agency, fulfillment, and satisfaction are enhanced [24]. Similarly, per attachment theory, experiences of felt security such as *safe haven* (a time when an attachment figure provided care of comfort) and *secure base* (a time when the support provided by an attachment figure allows one to take a risk one would not have taken otherwise) in our early relationships constitute the basis of later socioemotional health [25]. As such, moments of closeness recalled in an RS intervention include times when one supported (secure base) or comforted (safe haven) another person and felt deep positive emotions such as joy, peace, satisfaction, or love [24]. For CSPs, experiencing interpersonal connectedness when helping members of their community is a common occurrence [26], thereby underscoring the cultural congruence of this intervention. RS has the potential to prolong and enhance the attention given to these moments, helping CSPs lower stress and motivating them to continue engaging in their meaningful and necessary work.

Although RS has not previously been tested with CSPs or HCPs, other interventions rooted in positive psychology, such as the Web-based Implementation for the Science of Enhancing Resilience (WISER) intervention, have been found to decrease burnout and depression and support work-life integration in neonatal intensive care unit HCPs [27]. The WISER intervention includes 6 modules, 2 of which are titled “Three Good Things” and “Relationship Resilience” and refer to reflecting on 3 positive moments that occurred that day and reflecting on positive interactions, respectively. Considering RS’s focus on reflecting and enhancing positive emotions linked to a relational memory, there are considerable similarities with these 2 modules of WISER. Likewise, support for the RS intervention exists in its prior findings with other samples. Findings across studies with parent-child dyads, long-distance couples, older adults, and male adolescents in residential treatment have shown that RS can improve emotional states, psychological agency, relational schemas, and relationship satisfaction compared with control conditions [28-32]. Likewise, the RS intervention shows promise in affecting physical health by impacting health behaviors and lowering heart rate [29,31]. In addition, previous studies report that RS might be especially effective in HL populations because of its compatibility with cultural values [28]. Considering the potential of RS in positively impacting psychological and physical health in different populations, as well as evidence suggesting cultural computability, it is worth exploring its effect among HL CSPs.

In this study as well as in previous studies [26,28-32], while maintaining its core characteristics, the RS intervention is tailored to our priority population of HL-serving CSPs. This was done based on discussions held between the investigative team and the CSPs regarding what brings them meaning in relation to the job. Furthermore, the results of a qualitative study of *promotores’* experiences during the pandemic revealed important insights regarding the meaning derived from work as well as the unique challenges posed by working during the pandemic [17]. The challenges reported included enduring their own and their community’s losses, which placed them at risk of compassion fatigue or vicarious traumatization, along with feelings of grief and anxiety [17]. However, by being able to provide help to the community during difficult times, their work also provided a source of agency and meaning [17]. Therefore, in this investigation, RS focuses on assisting CSPs’ recall moments of connection with community members while doing their work and engaging with the community.

Finally, the participants in this study are all CSPs who serve the HL community, with most of them being HL themselves. Although we recognize that *Hispanic* and *Latinx* refers to individuals with origins in different countries, racial backgrounds, and levels of acculturation, there are cultural values that seem to be shared across communities of Latin American origin. Shared values include, among others, *simpatía*, the inclination toward emotional positivity or warmth and the avoidance of negativity or conflict with others, and *familismo,* valuing close family connections above the individual or self [33,34]. Considering that RS focuses on finding and reflecting on moments of warmth and connection, this intervention is culturally compatible with HL populations without the need for substantial tailoring [24]. In agreement, a previous study reported that HL mothers, but not non-HL mothers, showed a greater response to an RS intervention than to a personal savoring intervention [24]. Likewise, besides cultural congruence, HL CSPs share the fact that their job is caring for and supporting community members. In other words, CSPs’ day-to-day interactions with members of their communities may lead to various moments of connection that can be used throughout the RS intervention [26]. As such, the characteristics of their job may facilitate engagement with RS.

CSPs are required to support low-income HL communities’ health. If their physical and psychological well-being are at risk, their ability to reach community members through their programming is compromised. Considering the challenges endured in the pandemic and the risk factors associated with both their demographic characteristics and their job responsibilities, there is a need for interventions that support HL CSPs’ well-being. Bearing in mind this intervention’s compatibility with CSPs’ occupation and cultural background, RS holds immense potential.

### Objectives

This study aims to analyze the effects of an RS intervention on (1) HL CSPs’ cardiometabolic health (cardiometabolic risk and health behaviors) and (2) the risks of CSPs leaving the workforce. We hypothesize that compared with participants assigned to the waitlist control condition, those in the RS condition will exhibit the following health improvements:

- 1a. Exhibit lower cardiometabolic risk (lower heart rate variability [HRV]) during a discrimination stressor task
- 1b. Report more favorable cardiometabolic health behaviors pertaining to sleep, exercise, and diet
- 2a. Show reduced threats to CSPs leaving the workforce (greater work satisfaction and psychological agency and lower burnout)
- 2b. Exploratory hypothesis: That introduction of the RS intervention into agencies will result in greater engagement, as evidenced by the following: agencies will show increases in their demonstrated reach to community members through their health programing (ie, fewer absent days, more health-related events offered, larger community attendance at their events, and larger retention of community members in their programs) during the period following the introduction of RS as compared with the 6 months preceding the introduction of RS

## Methods

### Ethical Considerations

In conducting this study, careful attention was given to the ethical considerations that guide the treatment of research participants, ensuring the protection of their rights, privacy, and well-being throughout the study.

1. The study was conducted in accordance with the Declaration of Helsinki, and the protocol was approved by the University of California, Irvine, Institutional Review Board (UCI IRB #1596).
2. Informed consent: All participants gave their informed consent for inclusion before they participated in the study.
3. Privacy and confidentiality protection: All identifiable data collected were removed and replaced with a code. A securely maintained list links this code to the identifiable information, and is kept separately from the research data. All research data are maintained in a secure location at the University of California, Irvine, to which only authorized individuals have access. The data are stored electronically on a cloud and an external drive in an encrypted, password-protected file. Video or audio recordings are stored in a secure location; these data are transcribed and deidentified before analyses.
4. Compensation: The CSPs are compensated for their participation in the study, with compensation broken down as follows: US $30 for baseline visit 1, US $40 for baseline visit 2 (waitlist control group only), US $25 for each intervention session (total US $100), US $40 for posttreatment session, and US $50 for the follow-up visit. Overall, participants can earn US $220 or US $260 depending on the assigned group (experimental vs control). Participants can choose to receive the compensation via Venmo (PayPal, Inc) or as a physical or web-based gift card.

### Eligibility Criteria and Recruitment

All participants will be CSPs employed at partner community health agencies, including Latino Health Access (LHA), Dignity Health, and other agencies, to be recruited throughout our enrollment period. Participating agencies and CSPs will serve HL communities and will be located throughout the United States. Inclusion criteria include being aged ≥18 years and working (for payment or as a volunteer) as a CSP serving low-income HL families. Given that this pilot study is intended to test RS on a new population, we identified a sample size (n=80) that would allow us to identify a signal but not a statistically significant (P<.05) effect. This study aimed to generate a pilot study that could be used to support an adequately powered randomized controlled trial.

Participants will be recruited both offline and via email through their agencies, which will post advertisements in English and Spanish at their facilities, announce the opportunity in staff meetings and via email, and facilitate introductions between the research team and the CSPs. Interested participants will contact research personnel.

### Randomization and Overview

**Figure 1:**
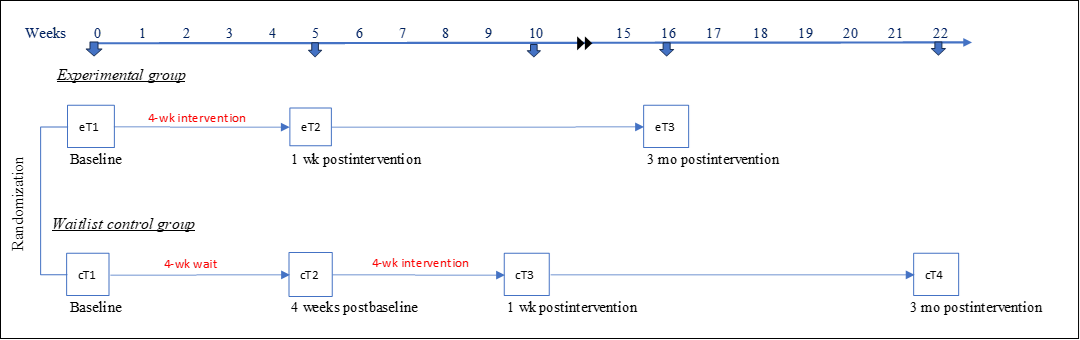
Randomization and timeline.

In this randomized controlled waitlist design (ClinicalTrial.gov identifier NCT05560893), the CSPs will be randomly assigned to an experimental group or a waitlist group. Both groups will complete an identical assessment battery (assessing health behaviors and psychological well-being) and complete stressor tasks (assessing cardiometabolic risk) before the intervention, after the intervention, and at a 3-month follow-up. The experimental group will receive the RS intervention (4 sessions, 1/wk) 1 week after the baseline assessment (Figure 1). The waitlist group will complete the baseline assessment, wait 4 weeks, complete a second baseline assessment, and 1 week later receive the RS intervention. After completing the RS intervention, the participants in both the experimental and waitlist groups will complete the postintervention assessment 1 week later and a follow-up 3 months later.

### Measures

#### Primary Outcomes

All the constructs and measures assessed are summarized in Table 1.

All assessments are available in both English and Spanish. Cardiometabolic health, as indicated by cardiometabolic risk and cardiometabolic health behaviors, will be the primary outcome and will be measured before the intervention, after the intervention, and at the 3-month follow-up. Cardiometabolic risk will be measured via HRV in response to a stressor task. The stressor task will consist of a discrimination stream-of-consciousness (SOC) task [35,36], where participants will talk for 4 minutes about an occasion where they felt discriminated against. Before completing the SOC task, participants will complete a questionnaire [36] that will provide them with a list of commonly occurring discrimination events from which they can select an event that has occurred to them. Vagally mediated HRV will be calculated from the variability of the interbeat intervals obtained from electrocardiogram (ECG) data. ECG data will be recorded using a Polar Verity Sense heart rate monitor or Movisens device, small wearable devices that the participants will place in their arm or chest, respectively, during the stressor task and at rest (before and after the stressor task baseline measures). Favorable cardiometabolic health behaviors include behaviors pertaining to sleep, exercise, and diet and will be assessed using the Pittsburgh Sleep Quality Index [37], the International Physical Activity Questionnaire–Short Form [38], the Food Behavior Checklist [39], the 36-Item Short Form Health Survey [40], and 2 self-developed scales (subjective assessment of overall health and impairment owing to overall health).

The measures and tasks used to assess the primary outcome, cardiometabolic health, are described in the following sentences:

1. Everyday Discrimination Scale: the participants will complete a Spanish version of the Everyday Discrimination Scale [36], a 9-item scale that assesses the frequency and the attribution of discrimination experiences (eg, In your day-to-day life, how often are you treated with less respect than other people are?) to assist them in identifying a discrimination experience to speak about in the SOC task. Items are rated on a scale of 0 (never) to 5 (almost every day), with higher scores indicating a higher frequency of discrimination experience. Previous studies have demonstrated good reliability and validity in HL samples [41].

2. SOC task: after a 2-minute baseline task (sitting with no stimuli), the participants will be asked to select an experience of discrimination to focus on for a SOC task. To help them do this, the participants will complete a brief questionnaire of common discrimination experiences, followed by a 5-minute interview to help them select among the experiences reported on the questionnaire. This cardiovascular reactivity task is modeled after a SOC task previously used in our laboratory [35], modified to be ecologically relevant to this population. The SOC task will ask the participants to speak for 4 minutes about the discrimination experience, focusing on the thoughts and feelings they had. The participants will then complete a 2-minute resting-recovery task wherein they will speak about a positive memory. The prompt for the SOC task is as follows (prompt translated to Spanish and available in English and Spanish, based on participant’s preference):

For the next four minutes, I would like for you to please discuss in detail your experience during this time that you have identified when you felt [insert discrimination experience here]. Please discuss your thoughts, feelings, hopes, and concerns about the experience. Remember, there are no right or wrong answers, so please say whatever comes to mind. Your job is to talk continuously about anything that comes to mind about this experience for the 4-minute period. We will record your responses because how you are thinking and feeling about this experience is very important to us.

3. Pittsburgh Sleep Quality Index: the participants will report on their sleep quality using the Spanish version of the Pittsburgh Sleep Quality Index [42], a 10-item measure that assesses sleep quality and duration. Items (eg, How many hours of actual sleep do you get at night—this may be different than the number of hours you spend in bed?) are rated on a scale of 0 to 3, whereby higher scores reflect more sleep disruption. Previous studies have demonstrated good reliability and validity in HL samples [43].

4. Food Behavior Checklist: the participants will report on their diet using the Food Behavior Checklist for Spanish speakers [39], a 16-item questionnaire that measures basic dietary behaviors, including the frequency of fruit, vegetables, juice, and low-fat dairy consumption; food insecurity; shopping; and cooking techniques. Previous studies have demonstrated good reliability and validity in HL samples [44].

5. Thirty six-Item Short Form Health Survey: the participants will report on their overall health using a Spanish version of the 36-Item Short Form Health Survey [40], a 36-item quality of life measurement that assesses 8 health-related domains, including physical functioning, role limitations because of physical problems, bodily pains, general health perceptions, vitality, social functioning, role limitations because of emotional problems, and perceived mental health. Previous studies have demonstrated good reliability and validity in HL samples [45].

6. Subjective assessment of overall health: the participants will report on their subjective assessment of their overall health by answering a 2-item scale designed for this study. Items assess perceptions of health (eg, Compared with men or women of your age, how would you define your health?) and are rated on a scale of 0 (bad) to 5 (excellent).

7. Impairment owing to overall health: the participants will report on impairments owing to their overall health by answering a 2-item scale designed for this study. Items assess behaviors related to health (eg, During the past 3 months, how many times have you missed school or work for physical or health problems?) and are rated on a scale from 0 (never) to 5 (>10 times).

8. International Physical Activity Questionnaire—Short Form: the participants will report on their exercise habits using a Spanish version of the International Physical Activity Questionnaire–Short Form [38], a 7-item scale that assesses the duration and intensity of physical activities undertaken over the previous 7 days, including the number of hours engaged in physical activity at different intensities, including walking, moderate-intensity activities, and vigorous-intensity activities.

**Table 1:** Outcomes: constructs and measures assessed.

Outcomes and constructs			Measures assessed
**Primary outcome: cardiometabolic health**			
	**Cardiometabolic risk**		
		Stressor-level heart rate variability	Everyday Discrimination Scale SOC^a^ task
	**Cardiometabolic health behaviors**		
		Sleep quality	Pittsburg Sleep Quality Index
		Diet	Food Behavior Checklist
		Overall health	36-Item Short Form Health Survey
		Subjective assessment of overall health	Self-Developed Scale
		Impairment due to overall health	Self-Developed Scale
		Exercise	International Physical Activity Questionnaire–Short Form
**Secondary outcomes**			
	**CSPs’^b^ threat to leaving the workforce (psychological well-being)**		
		Psychological well-being	Brief Symptom Inventory
		Emotion	Self-Assessment Manikin
		Closeness to community	Inclusion of Other in the Self Scale–Community Version
		Trauma history	Traumatic Life Events Questionnaire
		Burnout	The COVID-19 Burnout Scale Maslach Burnout Inventory
		Job satisfaction	Job Satisfaction Survey
		Psychological agency	Coder-rated measures based on SOC
		Reflective functioning	Coder-rated measures based on SOC
	**Intervention acceptability**		
		Savoring uptake	Self-developed questionnaires
		Intervention acceptability	Self-developed questionnaires
	**Community reach**		
		Number of days CSPs worked	Information obtained from agency
		Number of health-related events offered	Information obtained from agency
		Retention of community members in programs	Information obtained from agency
		Community attendance at events	Information obtained from agency

^a^SOC: stream-of-consciousness.

^b^CSP: community service provider.

#### Secondary Outcomes

Secondary outcomes include threats to CSPs leaving the workforce, which will be assessed via psychological well-being (ie, agency, burnout, and job satisfaction) and intervention acceptability. For these secondary outcomes, we will use the Brief Symptom Inventory-18 [46], the Self-Assessment Manikin [47], the Inclusion of Other in the Self Scale–Community Version (IOS-C) [48], the COVID-19 Burnout Scale [49], the Maslach Burnout Inventory [50], the Traumatic Life Events Questionnaire [51], and the Job Satisfaction Survey [52]. RS intervention acceptability and savoring uptake will be assessed via self-developed questionnaires. All these will be assessed before the intervention, after the intervention, and at the 3-month follow-up.

Finally, the CSPs’ delivery of cardiometabolic health programming to the community will be measured in an exploratory manner. This will be assessed via the number of days the CSPs worked, the number of health-related events offered, the retention of community members in the programs offered by the CSPs, and the number of community members attending the events. Data from the time following the introduction of RS will be compared with those from the 6 months preceding the introduction of RS. This information will be obtained from the health agencies’ institutional data. It is worth mentioning that these measures are not a reflection of the CSPs’ effectiveness, given that the CSPs may meet with fewer community members at any given time yet effectively support them in improving their health outcomes. Likewise, given different structures or job tasks across agencies (eg, leading groups, doing outreach, and follow-ups), there might be differences in how these measures are analyzed in the future. The collection of these data will not enable us to detect differences between the intervention and waitlist control groups. We intend to collect these data on the CSP’s delivery of health programming to lay the groundwork for a more in-depth future investigation of the impact of RS.

The measures and tasks used to assess the secondary outcomes are described in the following sentences:

1. Brief Symptom Inventory: the participants will report on their psychological well-being using the Spanish version of the Brief Symptom Inventory-18 [46], an 18-item measure assessing depression (eg, During the past week including today, how much were you distressed by feelings of worthlessness?), anxiety (eg, During the past week including today, how much were you distressed by nervousness or shakiness inside?), and somatization. Items are rated on a 5-point scale ranging from 0 (not at all) to 4 (extremely). Previous studies have demonstrated good reliability and validity in HL samples [53].
2. Self-Assessment Manikin: the participants will report on their emotions using the Self-Assessment Manikin [47], a widely recognized nonverbal pictorial measure that assesses emotional states, valence, and arousal levels using pictures to represent how a participant is feeling in response to stimuli.
3. IOS-C: the participants will report on closeness to the community using IOS-C [48]. This single-item pictorial measure uses Venn‐like diagrams—each diagram represents different degrees of overlap of 2 circles, progressing through 7 equal steps, representing lower and higher levels of closeness as the circles overlap more and more. The Inclusion of Other in the Self Scale has good test-retest reliability and convergent validity and has been used in samples with a large percentage of HL participants [54].
4. Traumatic Life Events Questionnaire: the participants will report their trauma history using the Spanish version of the Traumatic Life Events Questionnaire [51], a 23-item scale measuring whether an individual has experienced a wide range of traumatic events, including sexual assault, exposure to combat, or the unexpected death of a loved one, on a scale ranging from 0 (never) to 4 (≥5). Previous studies have demonstrated good reliability and validity in HL samples [55].
5. The COVID-19 Burnout Scale: the participants will report on their COVID-19 related burnout using the COVID-19 Burnout Scale [49], a 10-item scale that measures burnout feelings related to the pandemic. Items (eg, When you think about COVID-19 overall, how often do you feel hopeless?) are scored on a scale ranging from 0 (never) to 6 (all the time).
6. Maslach Burnout Inventory: the participants will report on their occupational burnout using a Spanish version of the Maslach Burnout Inventory [50], a 22-item scale that measures occupational exhaustion, depersonalization, and level of personal accomplishment related to work. Items (eg, I have become more callous to people since I have started doing this job) are scored on a scale ranging from 0 (never) to 6 (every day). Previous studies have demonstrated good reliability and validity in HL samples [56].
7. Job Satisfaction Survey: the participants will report on their job satisfaction using the Spanish version of the Job Satisfaction Survey [52], a 36-item assessment measuring attitudes related to the job and aspects of the job. Items (eg, I feel I am being paid a fair amount for the work I do) are scored on a scale ranging from 1 (disagree very much) to 6 (agree very much). Previous studies have demonstrated good reliability and validity in HL samples [57].
8. Savoring uptake: after completing the intervention, the participants will reflect on the savoring experience using the savoring uptake questionnaire that was developed for this study. The 6-item questionnaire measures the frequency at which participants have engaged in savoring since their last session, how useful they found savoring, how valuable they found savoring, whether they would recommend the program to others, and whether they would like to continue savoring on their own time. Items are scored on a scale ranging from 1 (not at all likely, useful, or valuable) to 5 (extremely likely, useful, or valuable).
9. Intervention acceptability: the participants will report on the acceptability of the RS intervention by completing the Relational Savoring Acceptability Questionnaire, a 14-item assessment created for this study to capture how participants feel before they begin the program (eg, I expect the program will improve my feelings about my work) and how they feel after they complete the program (eg, The program improved my health and well-being) on a scale ranging from 1 (strongly disagree) to 5 (strongly agree).

### Intervention

Consistent with prior work, the RS intervention will involve 4 weekly sessions, each lasting approximately 30 minutes, consisting of 2 phases: memory selection and memory reflection (Textbox 1).

**Relational savoring: intervention phases.**

**Phases and steps**

- Memory selection
  Recall a few memories of positive connectedness or attachment content when helping community members.
  Participants describe these memories and interveners ask follow-up questions designed to reveal the level of specificity, positivity, connectedness, and potential for spoiling in the memories.
  Interveners select one specific memory with the best content for the next phase.

- Memory reflection (5-step guided reflection)
  Sensory reflection (eg, Where were you? What did your surroundings look like? What could you hear?)
  Emotion reflection (eg, What did you feel?)
  Meaning making (eg, What did you think?)
  Future-oriented reflection (eg, What do you think will happen in the future thanks to this connection?)
  Mind-wandering reflection (eg, What else comes to mind?)

RS is a manualized intervention that has been adapted to allow CSPs the opportunity to savor memories of providing care to community members to enhance their sense of connectedness to the community [24,28]. In this study, as in previous studies, the intervention will be delivered by paraprofessionals (eg, undergraduate and postbaccalaureate students) [28]. Previous studies have shown that the intervention can be administered with high fidelity by paraprofessionals with a low level of clinical training (4 h of didactic training plus approximately 4 h of practice-based training) [28].

At the beginning of RS, the CSPs will complete a 1-minute mindfulness exercise, which is consistent with prior studies [28,29]. Next, during the first phase of the RS intervention (*memory selection*), the CSPs will be guided to generate memories of the times when they felt closeness, connection, or joy when they were able to support, encourage, or protect community members. The participant will be guided to select a memory with substantial attachment content (ie, secure base and safe haven memories). Alternatively, if the participant is unable to produce a memory explicitly related to safe haven or secure base content, they may select a memory involving a moment of positive connectedness or togetherness [28]. Then, during the second phase (*memory reflection*), the CSPs will engage in a 5-step guided process, where they will reflect on sensations, emotions, and cognitions associated with the selected moment. Sessions will be conducted via Zoom (Zoom Video Communications, Inc) by trained bilingual and bicultural research assistants in the participant’s language of choice (Spanish or English). The RS sessions will be recorded and transcribed to assess themes that emerge during the savoring sessions and to analyze therapist fidelity and therapist-client relationship quality. In addition, immediately before and after each intervention session, the participant will complete a 2-question survey assessing the participant’s mood (Self-Assessment Manikin) and perceived closeness to the community (IOS-C) [47,48].

### Data Analytic Plan

Descriptive statistics will be used to analyze the demographic characteristics of our sample population. A series of analyses of covariance will be conducted to examine the effects of RS on HRV, health behavior, psychological well-being, threats to CSPs leaving the workforce, and the reach of CSPs’ cardiometabolic health programming. We will compare outcomes at baseline, immediately postintervention, and at the 3-month follow-up across participants in the experimental versus waitlist group. HRV at baseline will be included as a covariate in analyses examining HRV, and all analyses will examine a range of potentially confounding covariates for potential use in analyses (eg, age, BMI, and socioeconomic status; those that are significantly associated with key study variables will be included as covariates in hypothesis testing). Effect sizes will be examined in all analyses. With a sample size of 80, it will be possible to detect small-to-moderate effects (yielding an effect size *d*=0.24) of RS on the CSPs’ cardiometabolic health, threats of CSPs leaving the workforce, and the delivery of cardiometabolic health programming. As stated in the *Eligibility Criteria and Recruitment* section, our goal is not to detect statistically significant effects but rather to look for effect sizes to inform future studies.

## Results

**Figure 2:**
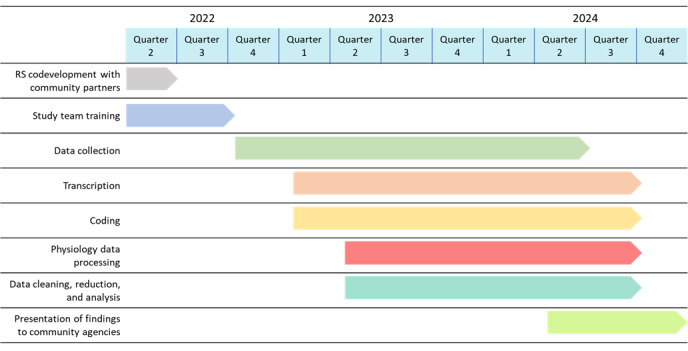
Study timeline. RS: relational savoring.

We expect that 80 CSPs will complete this study. The study was approved by the institutional review board in July 2022. CSPs from community agencies began participating in the study in October 2022, with 79 CSPs enrolled by November 2023. The data collection process is expected to continue until May 2024 and will include participants from LHA, Dignity Health, Green Madison Park, Orange County Health Promoters, and potentially from additional partner community agencies serving low-income HL communities. Transcription; coding; physiological data processing; and data cleaning, reduction, and analysis will occur throughout the following months and after the completion of data collection (Figure 2). We expect to publish our findings during the fourth quarter of 2024.

We will answer our central research questions using statistical analyses. In addition to these central analyses, we anticipate using qualitative methods to examine savoring sessions and discrimination SOC recordings for the themes that emerge (eg, nature of work, sources of strength or stress, and recurring topics). These qualitative analyses have the potential to reveal important information about HL CSPs, an understudied and important population.

## Discussion

### Expected Findings

This study will provide valuable information on the potential of RS to positively impact cardiometabolic health in HL CSPs and, indirectly, in the communities they serve. By improving cardiometabolic health behavior and lowering HRV, RS can reduce the risk of cardiovascular disease in CSPs. Simultaneously, supporting CSPs’ well-being and engagement can improve the community’s cardiometabolic health. Theoretically, if CSPs are healthy both psychologically and physically, their risk of leaving the workforce is reduced, and more community members will be reached and impacted by CSPs’ cardiometabolic health programming. This is particularly important in the context of communities’ recovery following the COVID-19 pandemic and considering the unique ability of CSPs to reach community members in ways that the health care system is unable to do. Therefore, RS interventions aimed at CSPs have the potential to influence well-being and cardiometabolic health at both individual and community levels.

Importantly, RS appears to be compatible with both HL CSPs’ cultural background and the characteristics of their job. This is evidenced by previous studies reporting greater benefits of RS in HL (vs non-HL) participants [28]. Likewise, CSPs are in constant interaction with community members, mostly in the context of CSPs providing some sort of support (eg, information, resources, and socioemotional support) [17,26]. This may facilitate engagement with the memories preferred for the RS intervention: moments where the participant helped or comforted others (safe haven) or when the participant provided support or encouragement (secure base) [24]. Congruence with both culture and occupation makes RS a particularly promising approach to support this population, especially when considering additional factors pertaining to the delivery of the intervention.

The use of culturally centered language is an important aspect to be considered in the delivery of psychosocial interventions [58]. As previously mentioned, the prompts generated for the RS intervention in this study were conducted in collaboration with our partner community health agency, LHA, to ensure culturally and linguistically appropriate wording. Relatedly, authors have contended that bilingual clinicians, rather than interpreters, are preferred to achieve optimal outcomes [58]. In this study, all participating CSPs are given the option of completing the intervention either in English or Spanish. Accordingly, RS is delivered by bilingual and bicultural trained paraprofessional interviewers, which may facilitate the use of culturally centered language throughout the interventions.

As previously mentioned, in this study, RS is delivered by paraprofessionals. Data from past studies have shown that RS can be administered at high fidelity by interveners who have had a low level of clinical training [28], such as interveners who are undergraduate college students and interveners who are *promotores* (trained community workers without formal education in mental health). This suggests that RS could be effectively implemented in settings without the need of professional health personnel, suggesting great promise as a highly scalable intervention. The data from this study will provide an opportunity to further examine RS intervener training, which could have important implications pertaining to this intervention’s potential for dissemination. The fact that we are delivering RS via telehealth further facilitates the interventions’ reach and potential impact. The flexibility offered by the remote setup reduces participant burden by avoiding a commute to and from a specific location, while broadening the timeframe in which the intervention can take place throughout the day both for participants and interviewers [59]. Likewise, this allows for participation throughout the country.

If RS is shown to be effective in supporting HL CSPs’ cardiometabolic health and psychological well-being, it could be implemented within the broader context of HL community health agencies’ personnel (employed and volunteer). In a more encompassing sense, once the CSPs have completed the intervention, they could potentially be trained as facilitators and conduct the intervention with community members. Although the cultural congruence between RS and HL values may boost its efficacy in our priority population, RS has the potential to support CSPs from other non-HL low-income communities. Medically underserved communities that rely heavily on community health agencies, such as those served by the CSPs participating in this study, may be particularly benefited by interventions such as RS that show great portability.

### Limitations

We anticipate that our study has limitations, some associated with the fact that the study will be conducted virtually, with participants usually at home or at their job location. Although remote sessions have advantages, it has been reported that it may be necessary to contact additional participants to reach enrollment and compliance goals in remote (vs in-laboratory) studies [60]. This is in addition to the fact that overall research recruitment has been affected since the pandemic. Likewise, the technology (devices, programs, and internet connection) needed to conduct remote studies can be unreliable, as has been found in previous studies [60]. Regarding the cardiovascular data, technical difficulties with the wearable devices and logistical issues in their delivery might result in missing ECG data, particularly given that the participants themselves will be tasked with deploying the devices. Given that one of the broader goals of the project is to provide information regarding the feasibility of the approach, any information yielded through this effort will be useful. However, missing data will preclude us from being able to understand the impact of RS on cardiometabolic health. Finally, we recognize that the planned analyses may be considered simple, yet this is what the sample size will afford. Furthermore, the data we can collect is limited to that which is feasible for the community agencies with whom we collaborate. These compromises accompany community-engaged work.

### Conclusions

Although considerable efforts are being made to expand the CSP model, it is necessary to ensure that the CSP workforce is adequately supported. The RS intervention holds immense potential in supporting HL CSPs’ overall health, specifically in lowering cardiometabolic risk. As such, RS could be used as a tool to promote resilience and support health in the growing number of CSPs. By supporting CSPs’ individual well-being, community health is strengthened. Our study will provide important contributions regarding the therapeutic effects of this psychosocial intervention in HL CSPs, who are particularly susceptible to cardiometabolic risks because of the COVID-19 pandemic, and consequently, on the communities they serve.

## Abbreviations

CSP: community service provider
ECG: electrocardiogram
HCP: health care professional
HL: Hispanic or Latinx
HRV: heart rate variability
IOS-C: Inclusion of Other in the Self Scale–Community Version
LHA: Latino Health Access
NHW: non-Hispanic White
RS: relational savoring
SOC: stream-of-consciousness
WISER: Web-based Implementation for the Science of Enhancing Resilience

## Data Availability

As data collection for this project is still ongoing, the complete data sets pertaining to this study are not yet available. Once the study is finalized, the data sets generated during this study will be available from the corresponding author upon reasonable request.
